# Combined transcriptomic and proteomic analysis reveals the response mechanisms of alfalfa to freezing stress

**DOI:** 10.3389/fpls.2025.1682825

**Published:** 2026-01-22

**Authors:** NaiPeng Ren, JieLin Liu, HongBao Wang, ZhaoMing Liu, XiangPing Liu, GuoLiang Li

**Affiliations:** 1 College of Agriculture, Heilongjiang Bayi Agricultural University, Daqing, China; 2 College of Animal Science and Veterinary Medicine, Heilongjiang Bayi Agricultural University, Daqing, China; 3 Grassland Research Institute of Heilongjiang Academy of Agricultural Sciences, Harbin, China; 4 Branch of Animal Husbandry and Veterinary of Heilongjiang Academy of Agricultural Sciences, Qiqihaer, China; 5 Heilongjiang Ecology Institute, Harbin, China

**Keywords:** alfalfa, freezing stress, molecular mechanisms, proteome analysis, transcriptome analysis

## Abstract

**Introduction:**

Alfalfa (*Medicago sativa L.*) is the most important perennial forage crop cultivated globally. However, extreme environmental conditions, such as freezing stress, can significantly impact alfalfa’s growth and development. The potential mechanisms through which alfalfa responds to freezing stress remain largely unexplored.

**Methods:**

In this study, we analyzed the physiological indices, transcriptomes and proteomes of the cold-tolerant alfalfa cultivar “Dongnong NO.1” and the cold-sensitive cultivar “Bara 218TR” at -5°C.

**Results:**

The results indicated that the levels of antioxidant enzyme and osmoregulatory substances in “Dongnong NO.1” were significantly higher than in “Bara 218TR”. Additionally, the levels of malondialdehyde (MDA) and relative electrolyte leakage (REL) were found be lower in “Dongnong NO.1” than in “Bara 218TR”. Further transcriptomic analysis revealed that the differentially expressed genes (DEGs) found in both alfalfa cultivars were predominantly enriched in the AP2/ERF-ERF transcription factor family and in multiple signaling pathways. Weighted gene co-expression network analysis (WGCNA) revealed that the physiological processes associated with freezing stress tolerance in the two alfalfa cultivars are closely linked to DEGs that regulate protein synthesis, calcium signaling, the inhibition of iron toxicity, and the reduction of cell wall stiffness. Proteomics analysis indicates that differentially abundant proteins (DAPs) respond to frost damage by maintaining protein stability, antioxidant defense, and metabolic regulation. Integrated transcriptomic and proteomic analyses indicate that pathways related to carbohydrate metabolism, biotic stress defense, cell wall modification, and phenylpropanoid biosynthesis are key to alfalfa’s response to frost damage.

**Discussion:**

This study improves our understanding of the molecular mechanisms underlying alfalfa’s freezing resistance and provides insights for the further screening and in-depth investigation of candidate genes with potential functions against freezing stress.

## 1 Introduction

Alfalfa (*Medicago sativa* L.) is a perennial herbaceous plant belonging to the Leguminosae family, often referred to as “the king of pasture” (Ma et al., 2022). It is highly resistant to cold and drought, and its cultivation area worldwide ranks second only to soybean (*Glycine max* L.). Alfalfa is widely distributed across various climatic zones, with a particularly high prevalence in temperate regions (Kulkarni et al., 2018). However, global climate change has led to the increasingly severe winters, with harsher weather conditions, making alfalfa vulnerable to cold or frost damage. This results in lower production performance and a significant decrease in overwintering power (D’Amours et al., 2023). This affects not only the yield and quality of alfalfa, but also causes significant losses to the sustainable development of animal husbandry. Therefore, freezing stress poses a serious threat to the alfalfa growth. Coping effectively with extreme climates and improving alfalfa’s tolerance to freezing have become urgent issues in the current research.

Low-temperature stress is a significant abiotic factor affecting crop yield and distribution. When exposed to low temperatures, plants respond to the cold environment through a series of physiological adaptation mechanisms aimed at maintaining normal growth and development (Wang et al., 2024). Membrane lipid response represents the primary mechanism through which plant cell membranes safeguard themselves against low-temperature stress (Liu et al., 2022a). Existing studies have indicated that the accumulation of unsaturated fatty acids and amino acids, together with the upregulation of the associated metabolic pathways, could act as effective strategies to enhance the cold tolerance of alfalfa (Li et al., 2022). Furthermore, in response to low-temperature stress, plants accumulate osmoregulatory substances to help them adapt to cold conditions. Research has shown that free proline (Pro) enhances alfalfa’s ability to withstand freezing temperatures by preventing rapid water loss. Soluble sugars (SS), including sucrose, fructose, galactose, and hydrosugar, sourced from the roots and stem of alfalfa, function as cryoprotectants, improving its freezing tolerance in cold and dry conditions (Xu et al., 2022). Another study showed that alfalfa’s soluble proteins (SP) change and synthesize specific antifreeze proteins in response to low-temperature stress. Cold tolerance is closely linked to the accumulation of cold-induced proteins (Sambe et al., 2015). Furthermore, low temperatures lead to the production of significant amounts of reactive oxygen species (ROSs) radicals in plants. ROSs accumulation is a primary factor that increases oxygen concentration, which can inhibit or damage plant growth (Qian et al., 2024). In response to low temperatures, plants produce antioxidant substances that scavenge ROSs, thereby adapting to temperature fluctuations (Baek and Skinner, 2012). Notably, peroxidase (POD) and catalase (CAT) activities have been reported to be higher in alfalfa prior to overwintering than after (Yao et al., 2023). Furthermore, enhanced superoxide dismutase (SOD) and ascorbate peroxidase (APX) activities contribute to improved frost stress tolerance in alfalfa (Wang et al., 2023a).

Low-temperature stress prompts plants to activate a series of molecular response mechanisms to adapt to and defend against cold environments. These mechanisms are triggered by the plant’s own cold-resistance genes, which encode specific proteins and molecules that enable plants to maintain their normal life functions under cold conditions. This is usually manifested as the co-expression of multiple genes, thereby improving the plant’s cold resistance (Rahman et al., 2024). Research has established relationships between *CBF1*, *CBF2 CBF3* and *ICE1*, *COR15A*, *COR47*, as well as candidate genes and other transcription factors (TFs) associated with low-temperature responses in plants. *ICE1-CBF* signaling pathway is recognized as the core regulatory module for cold acclimation and freezing tolerance enhancement (Chinnusamy et al., 2003). Furthermore, *COR* genes induce the expression of *CBF*, *MYB*, and other genes, including rice cold-responsive genes such as *OsCBF1*, *OsCBF2*, *OsCBF3*, and *OsMYB2*, indicating that *COR* regulates the expression of various genes to mitigate cold stress (Feng et al., 2022). Additionally, studies in rice (*Oryza sativa* L.) have identified that the *OsWRKY71*, *bZIP73^LAP^*, and *OsMYB30* genes positively regulate cold resistance (Kim et al., 2016; Liu et al., 2019; Lv et al., 2017). Eight *DREBs* from the AP2/ERF family, specifically *MsDREBs*, have been linked to long-term adaptation to freezing stress in the alfalfa cultivar “XinJiangDaYe” (Sheng et al., 2022). Another study of the same cultivar found that 15 *MsNACs* respond to cold, salt, and drought stresses (He et al., 2022). Some antioxidant enzyme genes also act as protective genes, helping plants to resist cold environments. For instance, the overexpression of *Fe-SOD* genes from *Arabidopsis thaliana* in alfalfa reduced the symptoms of secondary damage caused by winter temperatures and facilitated recovery from winter stress (McKersie et al., 2000). Another transcriptomic study in alfalfa found that genes associated with POD, SOD, and APX activities were activated by the application of salicylic acid (SA), providing resistance to freeze stress in alfalfa (Wang et al., 2023a).

In recent years, transcriptomes and proteomes analyses have played a significant role in identification and study of cold-resistant functional genes, as well as in elucidating the complex low-temperature stress response mechanisms of various plants, including *Arabidopsis thaliana* (Fowler and Thomashow, 2002), rice (Yun et al., 2010), and other plant species. In forage crop research, the integration of transcriptomic and proteomic analyses have been integrated to investigate variations genotype and stress tolerance in important crops such as ryegrass (*Lolium perenne* L.) (Pashapu et al., 2024), alfalfa (Hang et al., 2024), and maize (*Zea mays* L.) (Li et al., 2023), successfully elucidating a series of key regulatory modules. However, few studies have combined these two methods to analyze alfalfa’s frost resistance. In this study, we employed a combination of physiological, transcriptomic, and proteomic analyses to investigate the differences in the freezing response of alfalfa leaves from “Dongnong NO.1” and “Bara 218TR”, which exhibit different levels of cold tolerance. We aimed to explore the relationship between specific functional genes and alfalfa’s response to freezing stress. We examined the differences in the freezing response of alfalfa leaves between the two cultivars to identify functional genes that may affect the plant’s response to freezing stress, and to elucidate the underlying mechanisms. The findings of this study could be valuable for enhancing alfalfa’s resistance to abiotic stress and for breeding programs aimed at improving cold tolerance.

## 2 Materials and methods

### 2.1 Plant materials and freezing treatment

Based on the research group’s (Xuan et al., 2024) previous results on alfalfa cultivar introductions, two alfalfa cultivars were selected: “Dongnong NO.1” (D, cold tolerance genotype) and “Bara 218TR” (B, cold-sensitive genotype). All seeds were surface sterilized and placed on Petri dishes lined with double-layered sterile filter paper for germination at room temperature. After two days, seedlings with uniform growth were selected and transplanted into plastic pots containing a 1:1 mixture of nutrient soil and vermiculite. The potted alfalfa was then incubated at room temperature (25-27°C) with a photoperiod of 16/8 h. Three biological replicates were made for each cultivar, with seven seedlings retained in each pot. After 28 days of growth, the alfalfa seedlings were transferred to a low-temperature light incubator for a low-temperature exercise treatment at 4°C, with a photoperiod of 16/8 h, humidity of approximately 80%, and a light intensity of 12,000 lx for two days. The exercised seedlings were then cooled down in a low-temperature freezing incubator from 4°C to -5°C (3°C every 10 minutes), followed by a freezing treatment at -5°C for 5 hours 30 minutes. There were four treatment time points: 0 h, 2 h, 4 h, 6 h, followed by a two-day recovery period at room temperature. The treatment flow is shown in **Supplementary Figure S1**. The leaves of the “Dongnong NO.1” samples harvested at 0 h, 2 h, 4 h and 6 h after the start of the freezing treatment were labelled as D-0, D-2, D-4 and D-6, respectively. The leaves of “Bara 218TR” samples harvested at 0 h, 2 h, 4 h and 6 h after the start of the freezing treatment were labelled as B-0, B-2, B-4 and B-6, respectively. All samples were frozen in liquid nitrogen and stored at -80°C for later use.

### 2.2 Determination of phenotypic and physiological indicators

The frozen plants of the two alfalfa cultivars were returned room temperature for regrowth. The survival rate of each cultivar was counted after two days. Each pot contained seven plants of each cultivar, with three biological replicates per cultivar.


$$\text{Survival rate}=\frac{\text{Number of surviving alfafa plants}}{\text{Total number of alfafa plants}}\times 100\%$$

The relative electrolyte leakage (REL) assay was performed according to the previous method (Dionisio-Sese and Tobita, 1998) with slight modifications. 0.5 g of alfalfa leaves were weighed and placed in 30 mL of deionized water and vacuum infiltrated at room temperature for twelve hours. Initial conductivity (L1) was measured using a conductivity meter. The leaves were then boiled for 30 minutes, cooled to 25°C, and the conductivity (L2) was measured again.


$$\text{REL}=\frac{\text{L}1}{\text{L}2}\times 100\%$$

To determine the levels of five antioxidant activities, CAT, POD, SOD, APX, and glutathione reductase (GR), 0.1 g of fresh leaves were homogenized in phosphate buffer (50 mM, pH 7.0) and then centrifuged for 20 minutes at 4°C and 12,000 rpm.The resulting supernatant was used to detect the antioxidant activities. The CAT and APX activities were determined using an H_2_O_2_ decomposition assay (Melo et al., 2023). The POD activity was determined by a guaiacol oxidation assay (Piggott and Karuso, 2007). The GR activity was determined by NADPH colorimetric method (Ren et al., 2020). And the SOD activity was measured by using the SOD activity assay kit from Beijing Solarbio Science (China). All the test results were determined according to the manufacturer’s instructions.

The content of the osmoregulatory substance Pro was determined using the sulfosalicylic acid extraction method (Ren et al., 2020). The SP content was determined using the Kaumas Brilliant Blue method (Bradford, 1976), and the SS content was determined using the anthrone colorimetric method (Yu et al., 2022). The content of malondialdehyde (MDA) was determined using the thiobarbituric acid method (Draper et al., 1993).

### 2.3 RNA extraction and Illumina sequencing

Total RNA was initially extracted from leaf tissues of two cultivars using the RNA Easy Fast Plant Tissue RNA Extraction Kit (Beijing Tiangen Biochemical Technology Co., Ltd.), following the manufacturer’s instructions. The integrity and quality of the RNA were then assessed using agarose gel electrophoresis and the RNA Nano 6000 Assay Kit for the Agilent Bioanalyzer 2100 System (Agilent Technologies, CA, USA). RNA samples of acceptable quality (RIN value ≥ 6.5) were subsequently selected for constructing of cDNA libraries.

The mRNA was conjugated to oligo (dT) magnetic beads to enrich the eukaryotic mRNA. This was then mixed with Tris buffer and eluted at 80°C, after which a second conjugation was performed using Beads Binding Buffer. The mRNA was then fragmented into short pieces using Frag/Prime Buffer. The mRNA was then used as a template to synthesise both strands of cDNA, and splice and ligase enzymes were added sequentially to the PCR reaction product. The splice primers are listed in **Supplementary Table S1**. The ligation product was purified using the magnetic bead method. This was followed by double-stranded cDNA end repair, the addition of an A-tail and ligation of the sequencing junction. Fragment size selection was then performed using AMPure XP beads. Subsequently, PCR amplification was conducted according to the specified reaction conditions, and the amplified product was purified again using the aforementioned method to obtain the final cDNA library. Initial quantification of the library was performed using a Qubit 3.0 and the Qubit™ dsDNA HS Assay Kit, achieving a concentration of 1.5 ng/μL. Quality control was performed using a Qsep-400 to ensure that the peaks met the qualification criteria. The effective concentration of the inserted fragments was quantified via qRT-PCR, yielding a concentration greater than 2 nM. In this study, a total of 24 cDNA libraries were constructed, comprising six sample groups: D-0, D-2, D-4, D-6, B-0, B-2, B-4, and B-6, with three biological replicates per group. All samples underwent paired-end 150-bp sequencing using the Illumina NovaSeq 6000 platform. The sequencing results were stored in the FASTQ (fq) file format. Raw reads were filtered to remove junction reads and low-quality reads, resulting in high-quality clean reads. These clean reads were then aligned to the reference genome Medicago_sativa.Version3.genome.fa (https://figshare.com/articles/dataset/genome_fasta_sequence_and_annotation_files/12327602, accessed July 22, 2023) (Qi et al., 2024). Furthermore, all transcripts were annotated using the Nr, Nt, Swiss-Prot, Pfam, KOG/COG, KEGG, and GO databases (Zhang et al., 2021).

### 2.4 Differentially expressed genes screening and analysis

After normalizing the expression of each gene using Fragments Per Kilobase of transcript Per Million Fragments mapped (FPKM), difference analysis was performed between individual samples using DESeq2_edgeR (version 1.18.0). Two-way pairwise analysis was performed for all DEGs. Negative binomial distribution test (NB, Negative binomial distribution) was used to test the statistical significance of the differences in all reads. Finally, DEGs were filtered based on the results of fold change (FC) and false discovery rate (FDR). To ensure rigorous screening, the criteria FC ≥ 2.0 and FDR < 0.01 were selected to identify DEGs (Krishnamurthy et al., 2018). The statistical enrichment of DEGs in the kyoto encyclopedia of genes and genomes (KEGG) pathway was detected by KOBAS (version 3.0) (He et al., 2021). The metabolic pathways of the DEGs were analyzed by mapping them to the corresponding databases separately using the hypergeometric test (HGDT).

### 2.5 Weighted gene co-expression network analysis

Gene co-expression networks were constructed for all DEGs generated from the 24 samples using the WGCNA software package for R-Studio (version 3.3.0) (Lu et al., 2023). Highly correlated gene modules were identified based on expression levels generated by Salmon quantification. Modifications were partially made based on the previous study conducted by Yu et al. (2023): the expression threshold was set at 1, the module similarity threshold at 2, and the minimum number of genes per module at 30 for analysis. Subsequently, neighbor-joining matrices were constructed, and topological overlap matrix (TOM) similarity algorithm was applied to generate TOMs, which were then used for hierarchical clustering of single-gene modules in alfalfa. In order to analyze the correlation between modules and traits, the index data of CAT, POD, SOD, APX, GR, SS, SP, and Pro were correlated with the sequencing data of each module. Highly correlated modules were then identified and selected. Meanwhile, the hub genes of the key modules were screened based on gene KME values (Eigengene connectivity) using Cytoscape software (version 3.10.1), and visualized. Gene co-expression network analysis was then performed (Zhang et al., 2024).

### 2.6 Quantitative real-time reverse transcription polymerase chain reaction validation of transcriptome information

Consistency with RNA-Seq analysis data was verified using qRT-PCR on ten DEGs randomly selected from various subgroups: MS.gene82358, MS.gene008164, MS.gene00227, MS.gene37696, MS.gene00469, MS.gene62239, MS.gene02055, MS.gene80269, and MS.gene006165, MS.gene068509. qRT-PCR analysis was conducted using the CFX 96 Real-Time PCR Detection System (Bio-Rad, Hercules, CA, United States). Relative quantification of the three biological replicates was normalized to the internal reference genes, and the relative expression of the target genes was determined using the 2^-ΔΔCt^ method. The FC values were logarithmized in the RNA-Seq analysis, and the log_2_FC values were compared with the 2^-ΔΔCt^ values from qRT-PCR (Livak and Schmittgen, 2001). Information regarding all primers utilized in the analysis is provided in **Supplementary Table S1**.

### 2.7 Total protein extraction and proteolytic desalting

Total protein was extracted from the sample by TCA/acetone precipitation + SDT lysis (Zhu et al., 2023). Protein quantification was also carried out according to the manufacturer’s instructions of the Bradford Protein Quantification Kit from Thermo Fisher Scientific. Ten micrograms of each protein sample was tested for quality using 12% SDS-PAGE gel electrophoresis (Michaud and Asselin, 1995). At the end of the electrophoresis, the gels were stained with Kaumas Brilliant Blue R-250 staining, and destained appropriately to make the protein bands clear.

A total of 100 µg of protein was aliquoted, and DTT was added to achieve a final concentration of 10 mM. The mixture was incubated at 37°C for one hour. Once the samples had reached room temperature, IAM was introduced at a final concentration of 40 mM. The mixture was then maintained at room temperature, and shielded from light for 45 minutes. The samples were then diluted with ammonium bicarbonate, and the pH was measured to be 8. Trypsin was then added at a predetermined ratio of 50:1 (protein to trypsin), and the samples were incubated overnight at 37°C to facilitate the enzymatic reaction. The following day, 50 µL of 0.1% formic acid (FA) was added to the samples. The samples were then desalted to remove salts and other impurities using a C18 desalting column. The column was first activated with 100% acetonitrile (ACN) and then equilibrated with 0.1% FA. The treated samples were loaded onto the column and washed with 0.1% FA to eliminate unwanted impurities. Finally, the samples were eluted with 70% ACN, and the resulting solution was lyophilized into a powder and stored for future use.

### 2.8 Protein mass spectrometry analysis

The protein samples were separated using a RIGOL L-3000 high-performance liquid chromatography (HPLC) system (Beijing RIGOL Technology Co., Ltd). Mobile phase A consisted of 100% mass spectrometry-grade water containing 0.1% formic acid, and mobile phase B consisted of 80% acetonitrile containing 0.1% formic acid. Following 10 µL of phase A dissolving in the lyophilised powder, the sample was centrifuged at 4°C and 14,000 g for 20 minutes. Subsequently, 1 µL of the supernatant was utilized for liquid chromatography injection. After chromatographic separation, mass spectrometric analysis was conducted using a Q-Exactive mass spectrometer equipped with a Nanospray Flex™ ion source. The ion spray voltage was set at 2.4 kV, and the ion transfer tube temperature was maintained at 275°C. Mass spectrometry acquisition mode was data-dependent, with a scan range of 350–1550 m/z. The resolution of the primary mass spectra was 120,000 (at 200 m/z), with an automatic gain control (AGC) set to 3 × 10^6^ and a maximum injection time of 80 ms. The parent ions in the top 40 of the full scan were selected for secondary mass spectrometry, utilizing the high-energy collisional cleavage (HCD) method with a resolution of 15,000 (at 200 m/z), an AGC of 5 × 10^4^, a maximum injection time of 45 ms, and a collision energy of 27% for the peptides. The final generated mass spectral data were saved in.raw format.

### 2.9 Protein Identification and analysis of differentially abundant proteins

The raw data were identified and quantitatively analyzed using Proteome Discoverer 2.4 software for library search and comparison. Protein libraries constructed from cDNA libraries of the same batch of samples were utilized for database selection. The parameters for the analysis were as follows: enzyme: trypsin; fixed modification: carbamidomethyl; dynamic modifications: methionine oxidation (15.995 Da) and acetylation at the protein N-terminus; primary mass deviation: ± 15 ppm; secondary mass deviation: ± 0.02 Da; maximum allowed missed cleavage sites: 2; and a false discovery rate of less than 0.01.

The sample setup consists of four groups: D-0, D-4, B-0, and B-4, each with three biological replicates. The samples were grouped and analyzed, and the sets of differential proteins in each group were named using the convention “A_vs_B”, e.g. “B-0_vs_B-4” and “D-0_vs_D-4”. Proteins with FC > 2 and p < 0.05 were selected as the DAPs. KEGG enrichment analysis of DAPs was performed using KAAS (KEGG Automatic Annotation Server) software (Wang et al., 2021a).

### 2.10 Combined transcriptome and proteome analysis

All DEGs with the same and opposite expression trends were counted against DAPs. Subsequently, KEGG enrichment analysis was performed by comparing the background with all mRNA-protein relationship pairs using HGDT to identify significantly enriched pathways in mRNA-protein linkage pairs.

### 2.11 Statistical analysis

The data were analyzed using one-way ANOVA with SPSS software (SPSS Inc., Chicago, IL, USA, version 19.0) and tested for significant differences at the 5% level using Duncan’s method and Student’s t-test. The Origin 2018 software was used for plot analysis.

## 3 Results

### 3.1 Survival and physiological responses to freezing stress in two alfalfa cultivars

**Figure 1:**
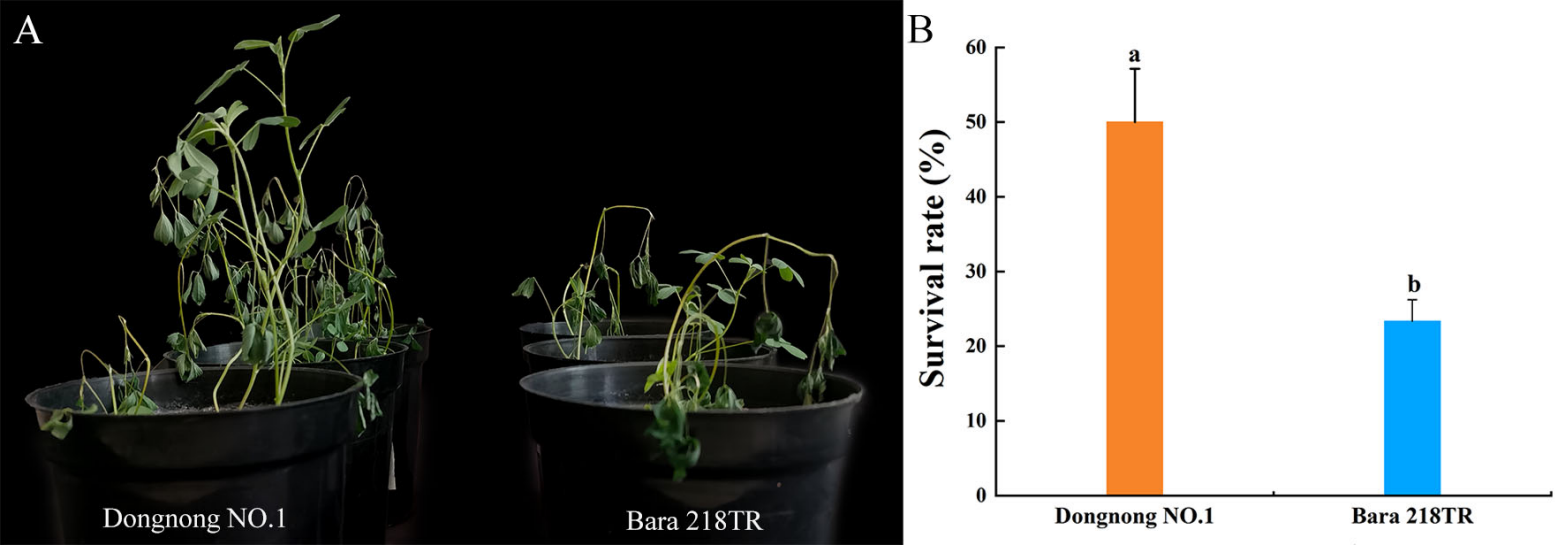
Phenotypes and survival rates of alfalfa cultivars “Dongnong NO.1” and “Bara 218TR” under freezing stress. **(A)** Phenotypes of alfalfa cultivars “Dongnong NO.1” and “Bara 218TR” after two days of recovery culture at room temperature. **(B)** Survival rate. Data are expressed as mean ± SD (n=3), and different letters above the bars indicate that the values of “Dongnong NO.1” and “Bara 218TR” are significantly different under different freezing treatments (*p* < 0.05, Student’s t-test).

After freezing the two alfalfa cultivars, phenotypic changes were observed after they were placed at room temperature (25°C-27°C) for two days. Phenotypic changes were observed in both alfalfa cultivars after freezing treatment. All the alfalfa cultivars were affected by freezing stress to some extent: the leaves lost water and wilted, and some leaves fell off and died. However, the degree of damage of “Dongnong NO.1” was significantly less affected than “Bara 218TR” (**Figure 1A**). For further evaluation, the survival rates of the two cultivars were determined. After freezing, “Dongnong NO.1” retained three to four surviving alfalfa plants, yielding a survival rate of 50%, while “Bara 218TR” retained only one to two plants, resulting in a survival rate of 23.33% (**Figure 1B**). This indicates that “Dongnong NO.1” is more tolerant to freezing stress than “Bara 218TR”.

**Figure 2:**
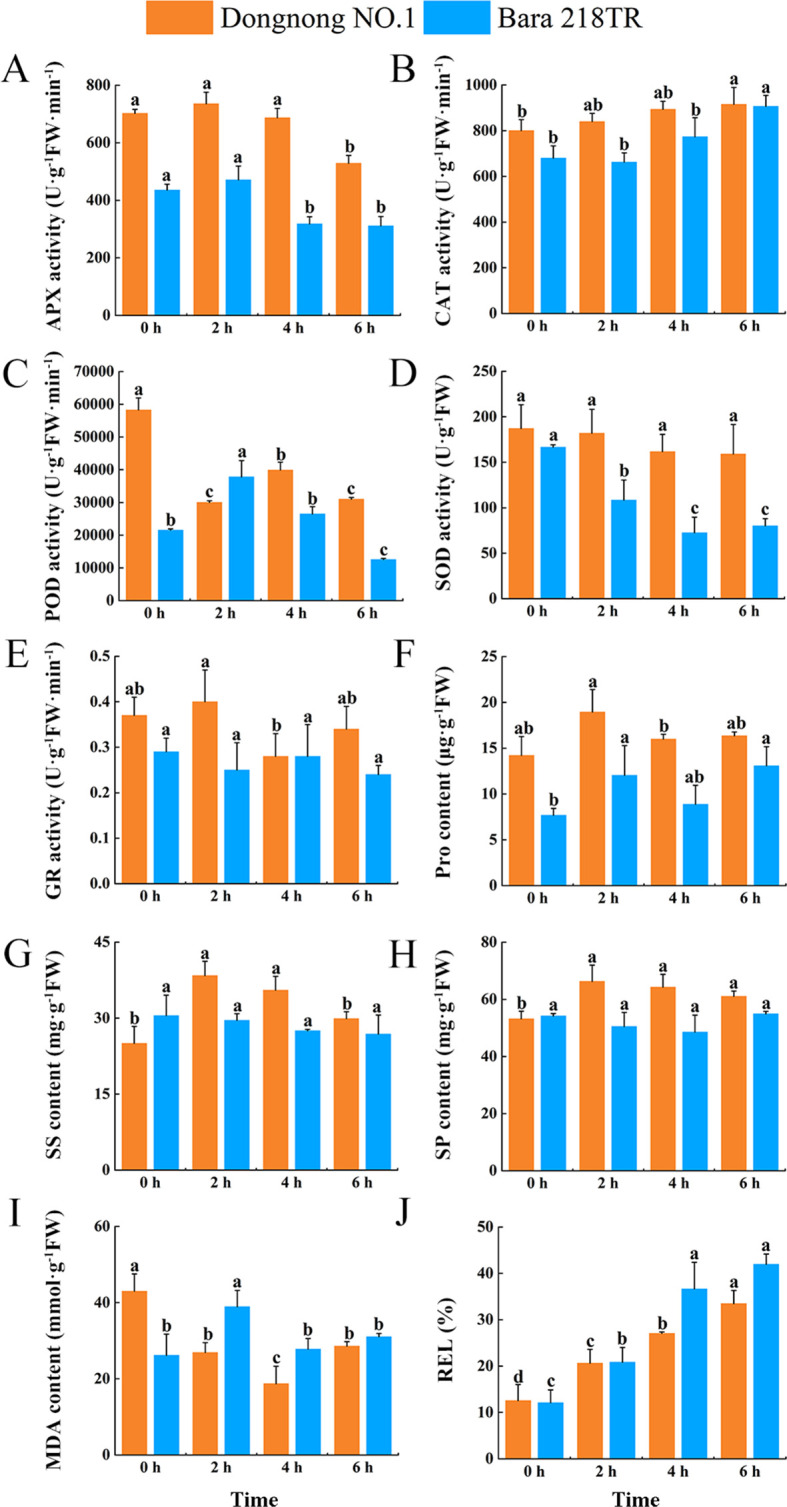
Physiological responses of leaves from two alfalfa cultivars to freezing stress. **(A)** APX. **(B)** CAT. **(C)** POD. **(D)** SOD. **(E)** GR. **(F)** Pro. **(G)** SS. **(H)** SP. **(I)** MDA. **(J)** REL. The data are expressed as mean ± SD (n=3), and the letters above the bar charts indicate significant differences (*p* < 0.05, Duncan's test) in the values of the two alfalfa cultivars under different freezing treatment durations.

To investigate the effects of freezing stress on physiology of alfalfa further, the antioxidant enzyme activities and osmoregulatory substance contents in two alfalfa cultivars were determined. As freezing stress time increased, the APX and GR levels of the two alfalfa cultivars peaked after two hours and then decreased. Overall, the levels of APX and GR were higher in “Dongnong NO.1” than in “Bara 218TR” (**Figures 2A, E**). The CAT activity of the two alfalfa cultivars increased gradually, while the SOD activity decreased slowly. However, the synergistic enhancement of CAT activity was more significant and higher in “Dongnong NO.1” than that in “Bara 218TR” (**Figures 2B, D**). The POD activities of the two cultivars showed opposite trends, and were significantly lower than those of “Dongnong NO.1” at all time points except two hours (**Figure 2C**). Pro content of “Dongnong NO.1” was highest at two hours and increased further at six hours, while proline accumulation was slower in “Bara 218TR” (**Figure 2F**). The peak SS and SP contents of “Dongnong NO.1” were also at the two-hour time point, being higher than those of “Bara 218TR” at the overall level after the stress (**Figures 2G, H**). This suggests that “Dongnong NO.1” initiated efficient antioxidant defense and osmoregulation at the early stage of freezing stress (two hours), delaying the accumulation of ROSs and membrane damage.

In order to investigate the effects of freezing stress on the cell membranes of alfalfa, the MDA content and the REL of the two cultivars were measured. The MDA content and REL showed the same trend over time, but the increment of MDA was lower for “Dongnong NO.1” (**Figure 2I**). The REL of “Bara 218TR” increased three and a half times at six hours compared with zero hours, while that of “Dongnong NO.1” increased only two and a half times (**Figure 2J**). This indicates that the membrane system stability of “Dongnong NO.1” was significantly better than that of “Bara 218TR”.

### 3.2 Transcriptome sequencing and *de novo* assembly of two alfalfa cultivars

After transcriptome sequencing, the raw reads generated from the initial screening were filtered to eliminate low-quality data, yielding 518,681,831 clean reads for assembly. This process generated 1,552,317,724 clean bases, with a GC content over 41% and a Q30 score exceeding 92% (**Supplementary Table S2**). All raw data have been deposited in the Sequence Read Archive (SRA) database of the National Center for Biotechnology Information (NCBI; https://www.ncbi.nlm.nih.gov/) under the accession number PRJNA1217929. For the clean reads, an average of 87.57% were successfully mapped to the reference genome, of which 40.78% were mapped to multiple loco and 46.79% to unique positions (**Supplementary Table S3**). Quality control results indicated that the sequencing data meet the required quality criteria rendering them suitable for subsequent transcriptome analysis.

### 3.3 Identification of DEGs and enrichment analysis of KEGG pathway

**Figure 3:**
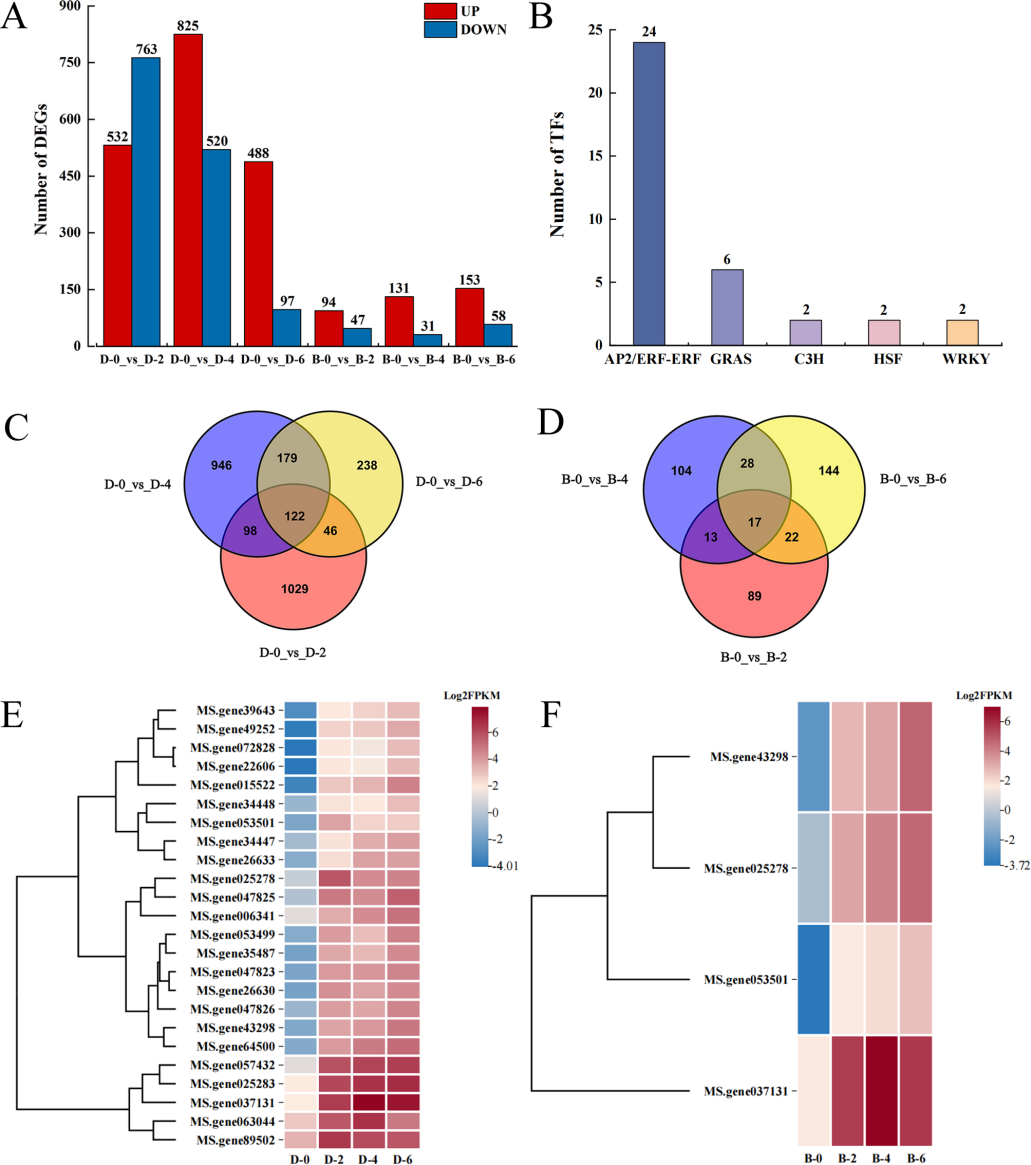
Freezing stress-responsive DEGs in two alfalfa cultivars. **(A)** Upregulated and downregulated DEG counts in different treatments. **(B)** Prediction of co-expressing TFs. **(C)** Venn plots of DEGs in “Dongnong NO.1”: 2, 4, 6 h vs. control (0 h). **(D)** Venn plots of DEGs of “Bara 218TR”: 2, 4, 6 h vs. control (0 h). **(E)** AP2/ERF-ERF TFs cluster heatmap in “Dongnong NO.1”. **(F)** AP2/ERF-ERF TFs cluster heatmap in “Bara218 TR”.

A comparative analysis of the DEGs was conducted. The results indicated that the number of DEGs identified in “Dongnong NO.1” was significantly higher than that in “Bara 218TR” at 2, 4, and 6 hours of treatment compared to the control (0 hours). Notably, the peak number of DEGs (1,345 in total) was detected at the 4-hour treatment (**Figure 3A**). Furthermore, 122 common DEGs were identified in “Dongnong NO.1” across all three time points, including 116 genes that were consistently upregulated and six genes that were consistently downregulated at the three time points (**Figure 3C**). In contrast, only 17 common DEGs were observed in “Bara 218TR” (**Figure 3D**).

The co-expressed DEGs constitute a core gene set shared across multiple time points, playing a crucial role in the sustained regulation of freezing stress responses. To identify key regulatory factors among these co-expressed DEGs, we predicted TFs. The AP2/ERF-ERF transcription factor family contained the highest number of co-expressed DEGs, with a total of 24 (**Figure 3B**). Cluster heatmap analysis showed that compared to the 0 h time point, the expression levels of AP2/ERF-ERF family TFs were upregulated in both alfalfa cultivars. Notably, four AP2 genes that were upregulated in “Bara 218TR” were also significantly upregulated in “Dongnong NO.1” (**Figures 3E, F**).

**Figure 4:**
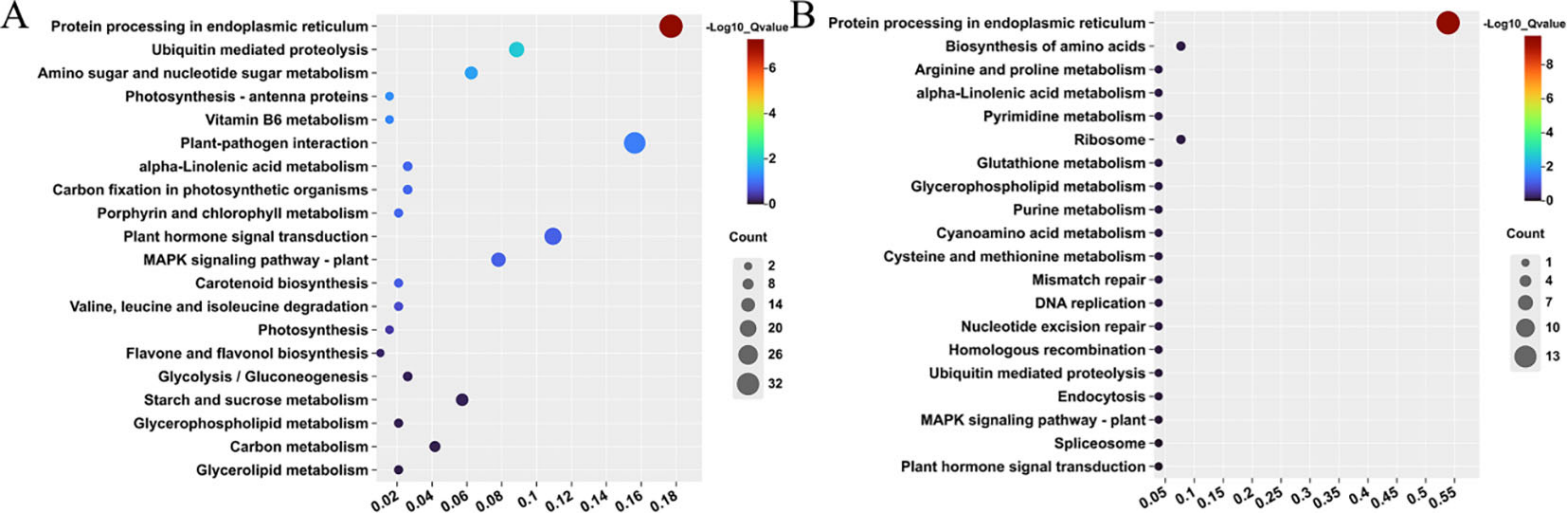
The top 20 KEGG pathways all DEGs in two alfalfa cultivars under freezing stress. **(A)** The top 20 KEGG pathways in “Dongnong NO.1”. **(B)** The top 20 KEGG pathways in “Bara 218TR”.

To further clarify the enrichment of DEGs, KEGG enrichment analysis was performed on the co-expressed DEGs across different time points in the two alfalfa cultivars. Significantly enriched pathways were identified by comparing with background genes, based on GeneRatio and Qvalue thresholds (**Supplementary Table S4**). The co-expressed DEGs of “Dongnong NO.1” were significantly enriched in five pathways, including “Protein processing in endoplasmic reticulum” (ko04141), “Plant-pathogen interaction” (ko04626), “Plant hormone signal transduction” (ko04075), “MAPK signaling pathway-plant” (ko04016), and “Ubiquitin mediated proteolysis” (ko04120). In contrast, the co-expressed DEGs of “Bara 218TR” were significantly enriched only in the “Protein processing in endoplasmic reticulum” pathway (ko04141) (**Figure 4**). These results indicate that “Dongnong NO.1” may regulate its response to freezing stress through multiple abiotic stress-related mechanisms, with a higher intensity of regulatory activation compared to “Bara 218TR”.

### 3.4 Analysis of DEGs involved in “Plant hormone signal transduction” pathways in alfalfa under freezing stress

**Figure 5:**
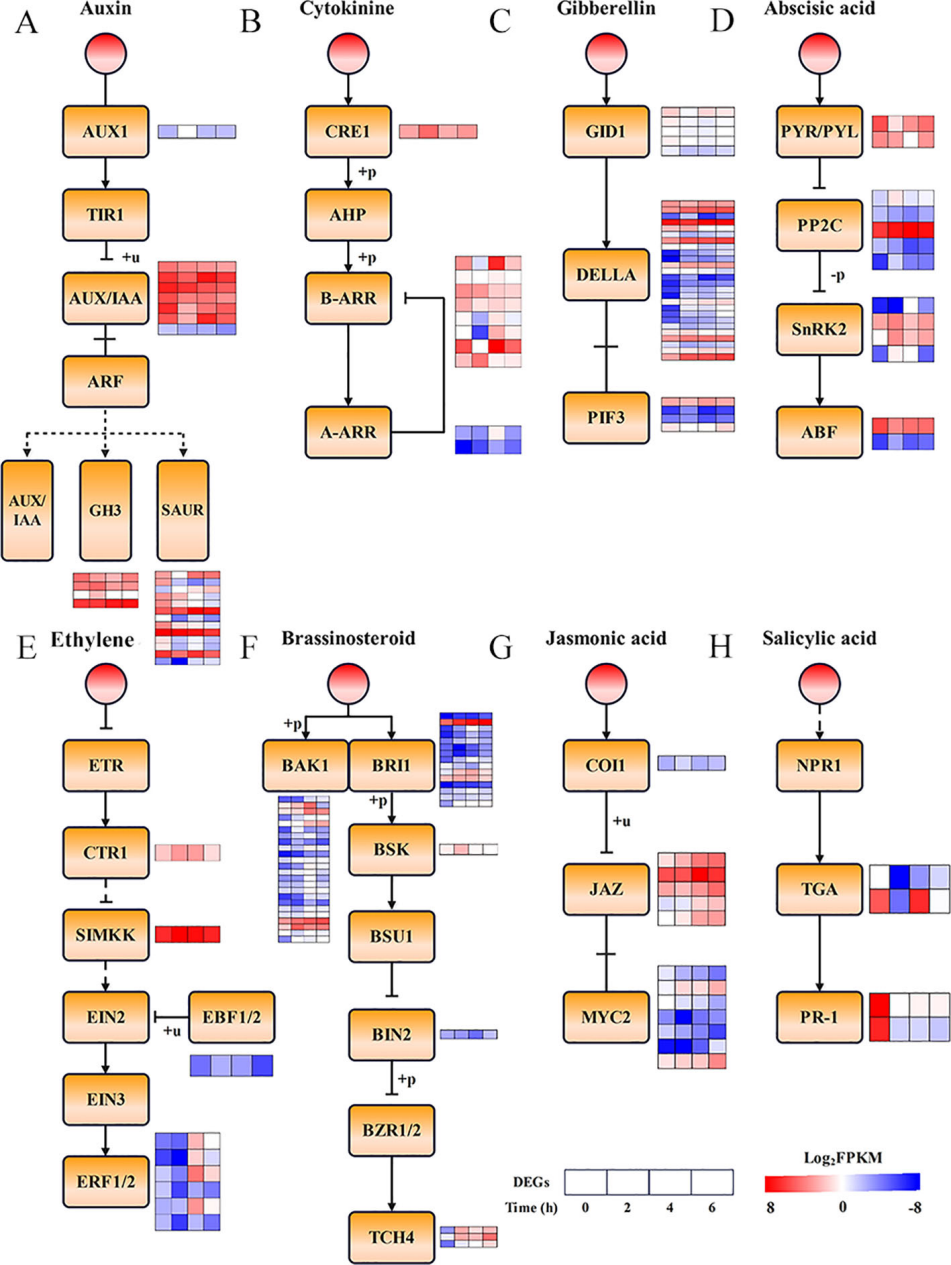
DEGs of the “Plant hormone signal transduction” pathway in alfalfa under freezing stress. **(A)** Auxin. **(B)** Cytokinine. **(C)** Gibberellin. **(D)** Abscisic acid. **(E)** Ethylene. **(F)** Brassinosteroid. **(G)** Jasmonic acid. **(H)** Salicylic acid. Four boxes indicate the Log2FPKM values for “Dongnong NO.1” at 0, 2, 4, and 6 hours.

In order to reveal the dynamic expression of DEGs involved in the phytohormone signaling pathway of alfalfa “Dongnong NO.1” under freezing stress, we depicted a phytohormone signaling pathway map by referencing the mapping resources from Kanehisa Laboratories. Within the auxin signaling pathway, one DEG encoding the AUX influx carrier protein *AUX1* was found to be upregulation at the initial stage of stress exposure (2 hours). Conversely, all seven *AUX/IAA* family genes showed significant downregulation in response to freezing stress. Correspondingly, their downstream target genes, *GH3* and *SAUR*, exhibited differential expression profiles as well, with four DEGs being upregulated and eleven DEGs being downregulated (**Figure 5A**, **Supplementary Table S5**). In the cytokinine synthesis pathway, the receptor gene *CRE1* was significantly upregulated at the initial stage of stress exposure. In contrast, six DEGs among the regulator *B-ARR* exhibited a downregulation trend while two showed an upregulation trend. Furthermore, the negative feedback regulator *A-ARR* indicated a trend of upregulation for both DEGs (**Figure 5B**, **Supplementary Table S5**). In the gibberellin synthesis pathway, the expression of all DEGs of the GA receptor *GID1* was downregulated, while nineteen DEGs were upregulated and seven were downregulated in the coregulator *DELLA*. One DEG was upregulated and three were downregulated in the downstream gene *PIF3* (**Figure 5C**, **Supplementary Table S5**). In the abscisic acid signaling pathway, two DEGs were significantly downregulated in the AbA receptor *PYR/PYL*; three DEGs were upregulated and two were downregulated in *PP2C*; three DEGs were upregulated and one was downregulated in *SnRK2*; and one DEG was upregulated and one was downregulated in *ABF* (**Figure 5D**, **Supplementary Table S5**). In the ethylene signaling pathway, the majority of DEGs expression of *CTR1*, *SIMKK*, *EBF1/2*, and *ERF1/2* was significantly upregulated under freezing stress (**Figure 5E**, **Supplementary Table S5**). In the oleuropein lactone signaling pathway, the expression of most DEGs of *BAK1*, *BRI1*, *BSK*, *BIN2*, and *TCH4* were also increased (**Figure 5F**, **Supplementary Table S5**). Eleven DEGs involved in jasmonate biosynthesis were significantly upregulated, including those encoding the COI1, JAZ, and MYC2 proteins (**Figure 5G**, **Supplementary Table S5**). In the salicylic acid biosynthesis pathway, all four DEGs encoding TGA and PR-1 were downregulated (**Figure 5H**, **Supplementary Table S5**). These results suggest that alfalfa adapts to freezing stress by dynamically regulating the multihormonal signaling network.

### 3.5 Construction of weighted gene co-expression network and gene mining of key modules

**Figure 6:**
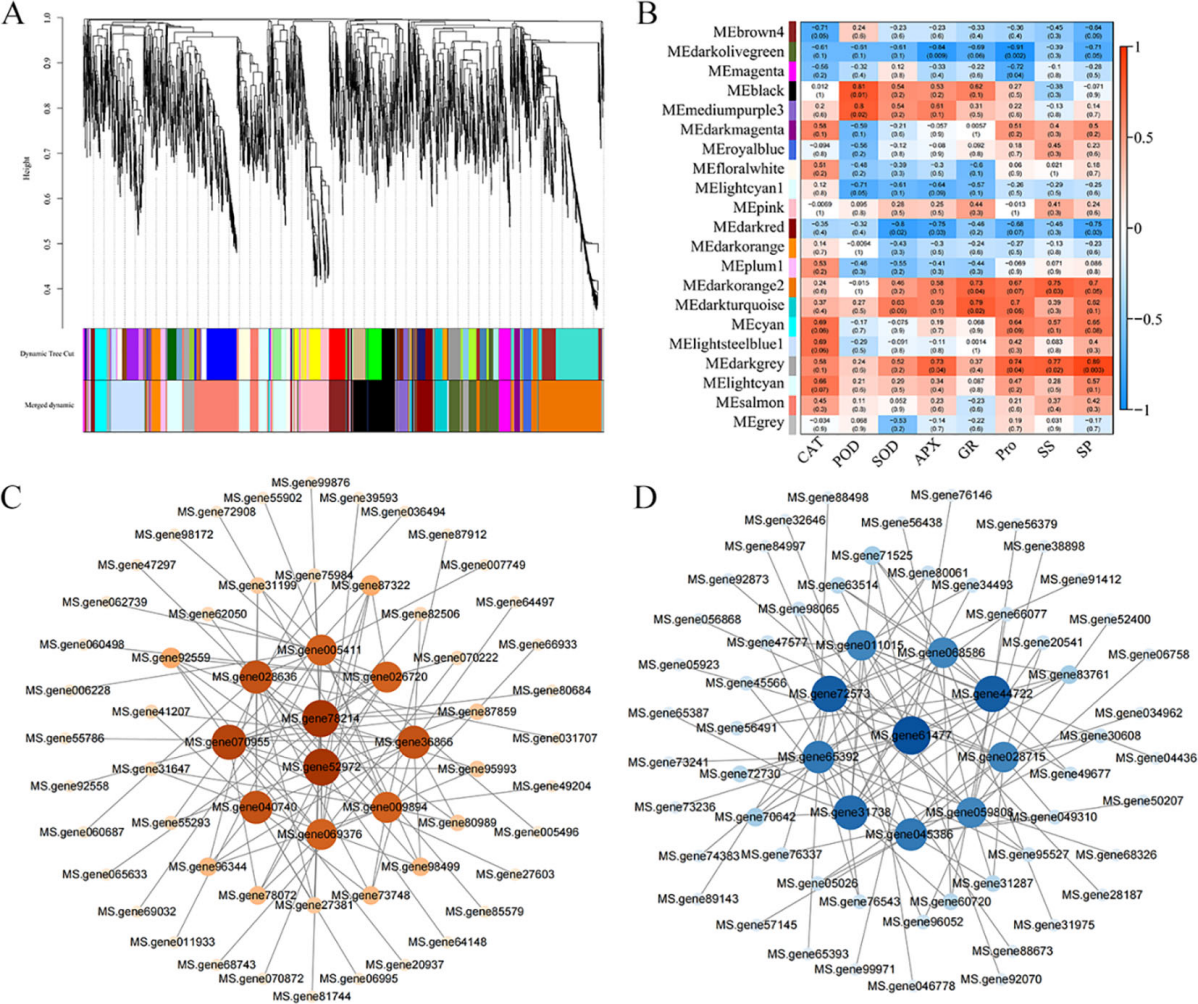
WGCNA analysis of two alfalfa cultivars under freezing stress. **(A)** Cluster dendrogram of 7,793 DEGs. **(B)** Heat map of correlation between twenty modules and physiological indicators. **(C)** Correlation network of the hub genes in darkgrey module and darkturquoise module. **(D)** Correlation network of the hub genes in darkolivegreen module and darkred module. Node sizes represent the level of intermediate degree centrality value: the higher the centrality value, the larger the node.

To further analyze the molecular mechanisms of the two alfalfa cultivars under freezing stress, an RNA-seq gene co-expression network was constructed. A total of 7,793 DEGs with clean data in different differential subgroups were selected for analysis, and a hierarchical clustering tree was constructed to generate twenty modules in total (**Figure 6A**). To investigate the correlation between the modules and different physiological indicators, the twenty modules were correlated with CAT, POD, SOD, APX, GR, Pro, SS, and SP. It was found that the darkgrey and darkturquoise modules were positively correlated with eight physiological indicators and the darkolivegreen and darkred modules were negatively correlated with eight physiological indicators (**Figure 6B**). To identify the candidate hub genes in the key modules, the KME values of all DEGs in the positively and negatively correlated modules were examined. The higher the value, the more important the gene was considered to be within its respective module. The genes with the top five KME values in each module were selected as candidate hub genes. The genes with the top ten gene-weight values in each surrounding correlation network were screened to construct the network map (**Supplementary Table S6**). Upon screening, it was found that the hub genes 60S ribosomal protein L14 (MS.gene52972) and protein sulfur deficiency-induced 1 (MS.gene78214), which are located in the positively correlated module, were induced to be upregulated and expressed by freezing stress. In addition, downstream genes such as cyclic nucleotide-gated ion channel 20 (*CNGC20*) (MS.gene27381), calcium-binding protein *CML45* (MS.gene96344), and chitinase 2 (MS.gene028636, MS.gene31199) were also enriched under freezing stress (**Figure 6C**, **Supplementary Table S6**). In the negatively correlated module, freezing stress induced the downregulation of expression of the hub gene monocopper oxidase-like protein SKS1 (MS.gene61477). The expression of metal-nicotianamine transporter *YSL7* (MS.gene45566) was down-regulated in the peripheral network (**Figure 6D**, **Supplementary Table S6**).

### 3.6 qRT-PCR validation

**Figure 7:**
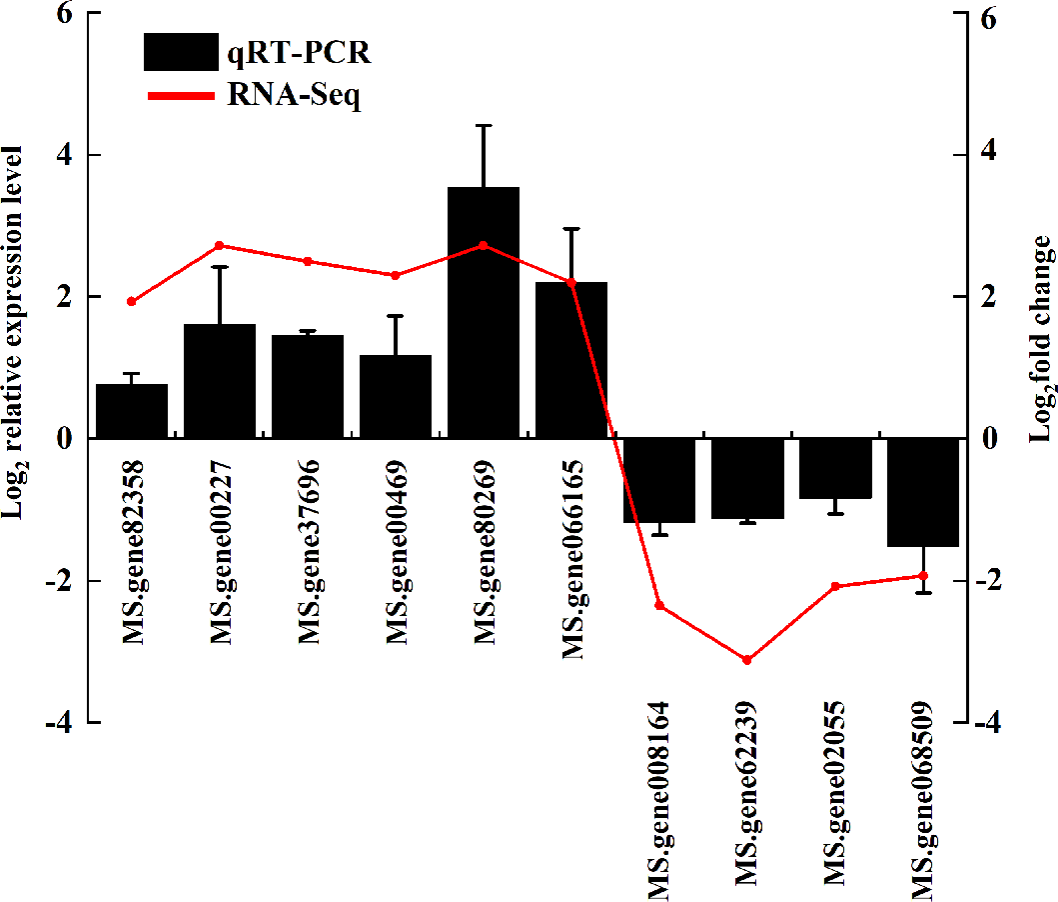
Comparison of RNA-seq and qRT-PCR results of ten DEGs under freezing stress. The RNA-seq results are expressed as Log_2_ (fold change). The qRT-PCR analysis of relative gene expression level data was calculated as Log_2_ (relative expression level).

To verify the accuracy of the RNA-Seq data, the expression levels of ten DEGs were determined using qRT-PCR (**Figure 7**). Six of the DEGs were found to be upregulated and four to be downregulated, demonstrating a consistent expression trend between the two analyses. This supports the reliability of the RNA-Seq results.

### 3.7 Analysis of DAPs in two alfalfa cultivars under freezing stress

**Figure 8:**
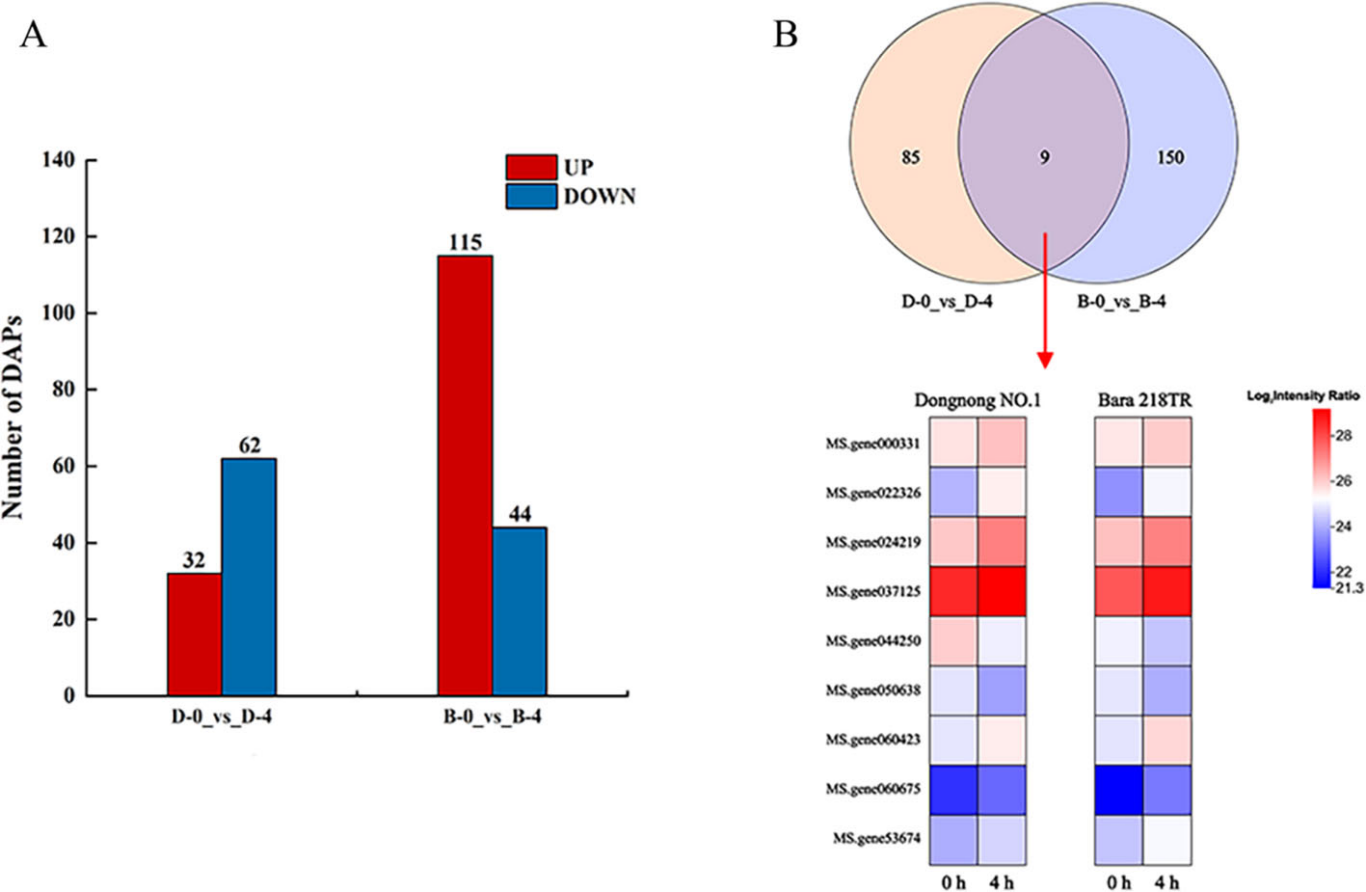
Freezing stress-responsive DAPs in two alfalfa cultivars. **(A)** Upregulated and downregulated DAP counts in two alfalfa cultivars. **(B)** Venn plots of DAPs between “D-0_vs_D-4” and “B-0_vs_B-4”.

To investigate DAPs in alfalfa under freezing stress, a total of 5,554 proteins were quantified using a label-free method. All the raw data have been uploaded to the iProX database (http://www.iprox.org) with the login number PXD060614. Compared with the control (0 hours), “Bara 218TR” produced a greater number of DAPs than “Dongnong NO.1” at 4 hours under freezing stress. The number of DAPs in “B-0_vs_B-4” was 159 (115 of which were upregulated and 44 of which were downregulated), compared with 94 DAPs for “D-0_vs_D-4” (32 of which were upregulated and 62 of which were downregulated). This indicate that “Bara 218TR” exhibits a stronger response to initial freezing stress than “Dongnong NO.1” (**Figure 8A**). In addition, “D-0_vs_D-4” and “B-0_vs_B-4” contained nine DAPs with the same expression trend, including seven upregulated DAPs such as chaperonin 10 Kd subunit, peroxidase and transketolase. There were two downregulated DAPs for copper amine oxidase, UDP glucuronosyl and UDP glucosyltransferase (**Figure 8B**). This suggests that DAPs in alfalfa can respond to freezing stress through quality control, antioxidant defense and metabolic support and repair.

### 3.8 Combined transcriptome and proteome analysis of two alfalfa cultivars under freezing stress

**Figure 9:**
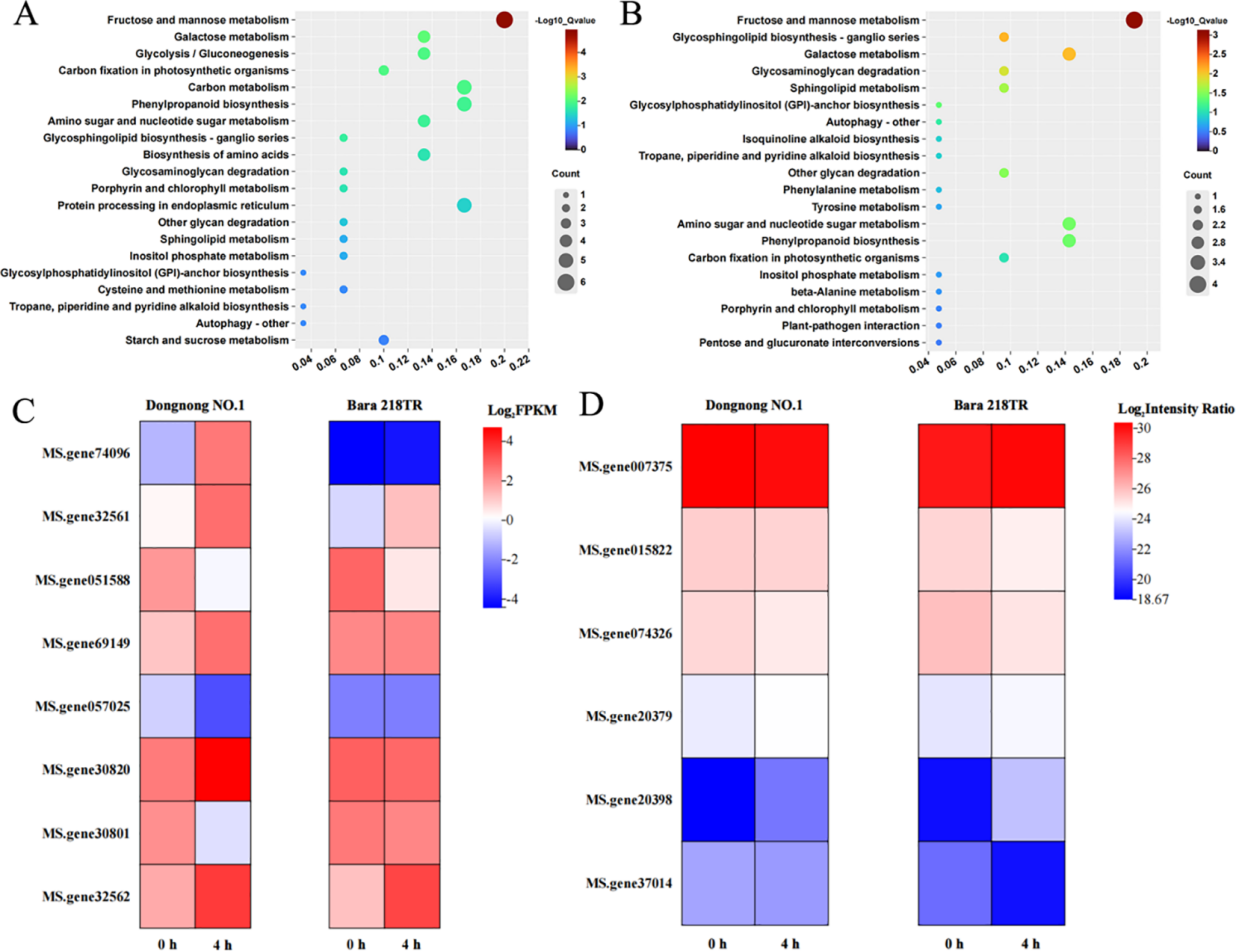
Top twenty enriched KEGG pathways for associated mRNA-protein junction pairs. **(A)** KEGG enrichment of DEGs. **(B)** KEGG enrichment of DAPs. **(C)** DEGs in the “Fructose and mannose metabolism” and “Galactose metabolism” pathways. **(D)** DAPs in the “Fructose and mannose metabolism” and “Galactose metabolism” pathways.

Comparison of proteomics and transcriptomics datasets reveals that ten mRNA-protein junction pairs in “D-0_vs_D-4” exhibit same expression trends, while thirteen pairs show opposite expression trends. In contrast, only seven pairs in “B-0_vs_B-4” had the same trend of expression (**Supplementary Table S7**). KEGG enrichment analysis of these DEG and DAP linkage pairs revealed that they were highly enriched in the “Fructose and mannose metabolism” (ko00051) and “Galactose metabolism” (ko00052) pathways (**Figures 9A, B**). These pathway contains three co-upregulated expression linkages pairs and two co-downregulated expression linkage pairs for sorbitol dehydrogenase (MS.gene30820-MS.gene20379), inducer-responsive protein 1 (MS.gene32561, MS.gene32562-MS.gene007375) and β-galactosidase 1 (MS.gene057025-MS.gene074326, MS.gene37014), among others (**Figures 9C, D**). In addition, the related DEGs were also significantly enriched in the “Carbon metabolism” (ko01200), “Phenylpropanoid biosynthesis” (ko00940), and “Glycolysis/Gluconeogenesis” (ko00010) pathways (**Figure 9A**, **Supplementary Table S8**). Similarly, related DAPs were significantly enriched in the “Amino sugar” (ko00940) and “nucleotide sugar metabolism” (ko00520) pathways (**Figure 9B**, **Supplementary Table S8**). These results suggest that the associated mRNA-protein linkage pairs respond to freezing stress mainly through sorbitol and galactitol synthesis, glycolysis/gluconeogenesis, flavonoid production and carbon metabolism.

## 4 Discussion

### 4.1 Physiological response of alfalfa to freezing stress

Several studies have reported the various effects of freezing stress on plant growth. These include the impairment of cellular structure and function, the activation of antioxidant systems, and the blockage of photosynthesis (Li et al., 2014, 2024). This study investigated the impact of freezing stress on the enzyme activities of antioxidant systems and the content of osmoregulatory substances in two alfalfa cultivars, with changes observed over the duration of freezing treatment. Overall, the physiological indices of “Dongnong NO.1” were higher than those of “Bara 218TR” (**Figures 2A–H**). It has been reported that different alfalfa cultivars exhibit varying capacities to accumulate osmoregulatory substances and demonstrate differing antioxidant system activities under adverse conditions, resulting in distinct stress resistance levels (Askari-Khorasgani et al., 2021). The findings of the present study therefore align with this conclusion, indicating that “Dongnong NO.1” exhibits greater resistance than “Bara 218TR”. Notably, the MDA content and REL of “Dongnong NO.1” were found to be lower than those of “Bara 218TR” across all measured levels (**Figures 2I, J**). MDA reflects the degree of lipid peroxidation of plant cell membranes, and the accumulation of MDA exacerbates cell membrane damage (Ashraf et al., 2021). REL reflects the degree of membrane permeability and damage, and increased membrane permeability leads to intracellular electrolyte extravasation, which elevates REL (Huang et al., 2015). A study on rice found that MDA accumulation in the resistant rice cultivar LJ25 was lower than in the cultivar LJ11, which is sensitive to low temperatures (Guo et al., 2022). Another study on alfalfa showed that dormant cultivars experienced significantly smaller changes in REL values after cold stress than non-dormant cultivars (Zhang et al., 2022). Consistent with the results of this study, these findings suggest that “Dongnong NO.1” experiences less cell membrane damage and is more resistant to freezing stress than “Bara 218TR”.

### 4.2 Analysis of KEGG pathways involved in DEGs induced by freezing stress in alfalfa

In order to elucidate the mechanism by which alfalfa responds to freezing stress, we identified 184,290 genes in “XinJiangDaYe” alfalfa reference genome using RNA-seq. and constructed ten comparisons by two-by-two pairing, with a total of 7,793 DEGs identified (**Figure 3A**). The number of acquired genes was basically the same as the number of DEGs in previous studies on low-temperature tolerance in plants (Cheng et al., 2018; Bhattarai et al., 2021). DEGs co-expressed by “Dongnong NO.1” and “Bara 218TR” were significantly enriched in the AP2/ERF-ERF transcription factor family. This family plays a key role in the plant’s response to frost (Liu et al., 2022b), with multiple members showing consistent upregulation (**Figures 3E, F**). This suggesting that AP2/ERF-ERF family may be crucial in alfalfa’s response to cold. Additionally, the co-expressed DEGs of “Dongnong NO.1” and “Bara 218TR” were mainly enriched in the “Protein processing in endoplasmic reticulum” pathway (**Figure 4**, **Supplementary Table S4**). Previous studies have shown that some genes encode molecular chaperones and fold proteins such as enzymes to maintain the normal function of proteins and relative stability of the intracellular environment, thereby enhancing plant tolerance to environmental stresses (Gupta and Tuteja, 2011). For instance, DnaJ and chaperone proteins in the Hsp70 and Hsp20 families help proteins to fold correctly or undergo endoplasmic reticulum-associated degradation (Yu et al., 2019). Moreover, glycosylation modifications in the endoplasmic can also increase protein stability, making them more resistant to denaturation induced by low emperatures (Strasser, 2018). Our study found that this pathway contains a large number of Hsp family chaperone proteins, such as 18.1 kDa class I heat shock proteins, 17.9 kDa class I heat shock proteins, and so on. Protein glycosyltransferase subunit 1B was induced to be upregulated in the comparison of two alfalfa cultivars for four hours of freezing treatment,. This is consistent with the results of previous studies and suggests that these genes may be involved in the plant’s response to freezing stress, improving alfalfa’s freezing resistance.

In “Dongnong NO.1”, co-expressed DEGs were highly enriched in “Plant-pathogen interaction”, “Plant hormone signal transduction”, “MAPK signaling pathway-plant” and “Ubiquitin mediated proteolysis” pathways (**Figure 4**, **Supplementary Table S4**). In European oilseed rape (*Brassica napus* L.), transcripts that were differentially expressed in the “Plant-pathogen interaction” and “Plant hormone signal transduction” pathways were significantly enriched under cold and freezing stress (Pu et al., 2019). Additionally, the MAPK signaling cascade pathway serves as a central regulatory hub in the plant’s cold stress response (Xing et al., 2024). Previous studies have indicated that the *MEKK1-MKK4/5-MPK3/6* signaling module regulates the expression of relevant cold-resistant genes within the nucleus of plant cells (Movahedi et al., 2025). In this study, we identified that one *MEKK1* gene, one *MKK4* gene, and three *MPK3* genes were significantly upregulated among the DEGs of “Dongnong NO.1” (**Figure 4**, **Supplementary Table S4**). This suggests the presence of a *MEKK1-MKK4-MPK3* signaling module in cold-tolerant alfalfa cultivars. The plant ubiquitin-proteasome system can respond to low-temperature stress by regulating protein stability. Previous study indicated that the rice E3 ubiquitin ligases *OsPUB2* and *OsPUB3* play a role in regulating cold stress (Byun et al., 2017). In our research, we identified an E3 ubiquitin-protein ligase PUB22, that was upregulated (**Figure 4**, **Supplementary Table S4**), which may serve a similar function.

### 4.3 Response to phytohormone signaling in alfalfa under freezing stress

Phytohormones are organic compounds produced in trace amounts by plants that play a crucial role in regulating their physiological processes, particularly in response to low-temperature stress. Research indicates that winter wheat (*Triticum aestivum* L.) Dn1 can respond effectively to low-temperature stress through phytohormone signaling (Wang et al., 2023b). The phytohormone signaling system in peppercorns (*Zanthoxylum bungeanum* Maxim.) exhibits complexity under low-temperature conditions, with notable differences in the response patterns between cold-tolerant and cold-sensitive cultivars (Tian et al., 2022). In this study, we found that *PYL*, *PP2C*, *SnRK2*, and *bZIP*, the core components of the ABA signaling pathway, were widely and significantly regulated in the abscisic acid biosynthesis signaling pathway, especially at the 2-hour and 4-hour freezing treatments in “Dongnong NO.1” (**Figure 5D**, **Supplementary Table S4**). A previous study in cucurbits (*Lagenaria siceraria* Standl.) reported the functional characterization of *PYL-PP2C-SnRK2s* in response to cold stress (Iglesias-Moya et al., 2024). Whereas bZIP TFs have been shown to respond to abiotic stresses via an ABA-dependent pathway (Banerjee and Roychoudhury, 2017). The present study identified an induced upregulation of a bZIP factor in a 2-h freezing treatment of “Dongnong NO.1”, which in turn drove the expression of ABA-responsive genes. Genes involved in the ethylene signalling pathway, including *CTR1*, *SIMKK*, *EBF1*/*2* and *ERF1*, were found to be upregulated in response to freezing stress. Furthermore, several studies have demonstrated that *ERF1B* expression can be increased via ethylene signalling to enhance cold tolerance (Zhao et al., 2025). Genes involved in the ethylene signaling pathway, including *CTR1*, *SIMKK*, *EBF1/2*, and *ERF1*, were found to be upregulated in response to freezing stress. This study found synchronised upregulation of four *ERF1Bs*,indicating that ethylene signaling peaks during stress at this time (**Figure 5E**, **Supplementary Table S5**). Ethylene and jasmonic acid are commonly reported to synergistically regulate abiotic stress responses in plants (Kazan, 2015), and the *COI1-JAZ-MYC2* complex is involved in cold signaling (Wang et al., 2019). JA treatment has also been shown to mediate tethylene and sugar metabolism in peach and enhance their cold tolerance (Zhao et al., 2021). This study found that early activation of *COI1* upregulation occurred in the jasmonate signaling pathway. Meanwhile, mid- to late-phase JA signaling was inhibited by *JAZ* accumulation, which induced *MYC2* gene expression (**Figure 5G**, **Supplementary Table S5**). These findings illustrate the synergistic regulation of the two signaling pathways. In the oleoresin lactone signaling pathway, BR binds *BRI1* and then interacts with and phosphorylates *BAK1*, which recruits *BSK* and allows for its continued phosphorylation, initiating BR-responsive genes that regulate stress resistance (Mao and Li, 2020). This study identified upregulated *BAK1*, *BRI1* and *BSK*, suggesting the involvement of BR signaling in the freezing stress response in alfalfa (**Figure 5F**, **Supplementary Table S5**).

### 4.4 Characterization of DEGs associated with antioxidant and osmoregulation in alfalfa under freezing stress

Ribosomes are key organelles for protein synthesis in cells, and their functional status directly affects the proteome remodeling ability of plants under low-temperature stress, which in turn regulates its freezing tolerance (Fakih and Germain, 2025). Previous investigations have demonstrated that the expression level of ribosomal protein P0 in potato (*Solanum tuberosum* L.) leaves is significantly upregulated under low-temperature stress, enabling the plant to adapt to the environment (Li et al., 2021). Ubiquitin-associated ribosomal proteins function as pivotal genes in the cold stress response of alfalfa (Pu et al., 2024). In the present study, WGCNA was employed to identify ribosomal protein L14 (*RPL14*) as a hub gene within the positively correlated module. This gene was found to be significantly upregulated under freezing stress, thereby implying its potential regulatory role in mediating plant freezing tolerance. Sulfur deficiency-inducible protein 1 (*SDI1*) is a key regulatory protein that is strongly induced in plants in response to sulfur deficiency. A previous study isolated the *SDI1* gene in the drought-tolerant sunflower (*Helianthus annuus* L.) and found that drought-tolerant sunflower had a higher level of *SDI1* transcripts than the drought-sensitive sunflower in the absence of water (Ouvrard et al., 1996). Our study found that the pivotal gene *SDI1* in the positive correlation module was induced by freezing stress and upregulated its expression. This suggests that *SDI1* may also be subject to freezing conditions in response to stress tolerance. Ca^2+^ as an important signaling molecule participates in the complex signaling process of plant cold stress. In *Arabidopsis*, the Ca^2+^ osmotic channel *CNGC20* positively regulates freezing tolerance by mediating cold-induced Ca^2+^ inward flow (Peng et al., 2024). Meanwhile, freezing stress caused changes in intracellular calcium ion concentration, which act as signaling molecules. These in turn induced the expression of *CaM/CMLs*, which interact with downstream target proteins to trigger cellular responses (Wu et al., 2023). It was found that *CsCML14*, *CsCML50*, *CsCML65* and *CsCML79* in chrysanthemum in response to low temperature and salt stress (Fu et al., 2022). In the positive correlation module, we observed the upregulation of the peripheral network genes *CNGC20* and *CML45*, which suggests that Ca²^+^ signalling is involved in the transcriptional regulation of alfalfa in response to freezing stress and the expression of downstream target genes (**Figure 6C**, **Supplementary Table S6**). This result is consistent with previous findings in alfalfa (Wang et al., 2022; Du et al., 2021), indicating that freezing stress rapidly activates calcium signaling pathways.

Monocopper oxidase catalyzes the oxidation of phenolic substrates, promoting cell wall lignification and cross-linking to enhance mechanical strength (Liu et al., 2021). In this study, we found that the *SKS1* gene, which is pivotal in the negative correlation module, was downregulated. This may be due to the contraction of protoplasts under freezing stress. The over-hardening of the cell wall may impede the contraction and exacerbate mechanical damage. Furthermore, upregulation of *SKS1* alleviated the stiffness of the cell wall caused by excessive lignification. The YSL transporter protein family plays an important role in the transport and distribution of metal ion in plants. Some studies have found that *YSL* genes are highly sensitive to iron deficiency in rice. For example, *OsYSL8* and *OsYSL9* showed a significant increase in gene expression after iron application (Saleem et al., 2024). In *Medicago truncatula*, *MtYSL3* was found to be involved in transporting iron and zinc to nitrogen-fixing nodules via the vascular system (Castro-Rodríguez et al., 2021). However, excessive iron ions in plant cells may produce a large amount of reactive oxygen species through the Fenton reaction, causing oxidative damage to cells (Berni et al., 2019). This study found that *YSL7* is negatively regulated, which may help to avoid the excessive accumulation of iron ions and alleviate the freezing damage to alfalfa cells by downregulating its expression to reduce the transport of iron ions under freezing stress (**Figure 6D**, **Supplementary Table S6**).

### 4.5 Response of DAPs in alfalfa to freezing stress

The cold-tolerant cultivar “Dongnong NO.1” exhibits a greater number of DAPs compared to the cold-sensitive cultivar “Bara 218TR”. This may be because “Dongnong NO.1” maintains elevated levels of key protective proteins even under non-stress conditions, whereas “Bara 218TR” relies more heavily on the expression of these proteins following cold induction. This demonstrates that the two alfalfa cultivars have different molecular response strategies to freezing stress. Plants usually prioritize the maintenance of protein function and defense against oxidative damage as a major survival response in an adverse environment (Kerchev and Van Breusegem, 2022). In this study, we found that the 10Kd subunit of chaperone protein was upregulated for expression with Clp protease, which has been shown to prevent protein misfolding and aggregation. Clp protease also degrades irreversibly damaged proteins and complements chaperone protein function (Sun et al., 2021; Dong et al., 2021). The synergistic upregulation of the two proteins suggests that maintaining the stability of the protein mass-control system is a central strategy for frost resistance in alfalfa. Freezing stress induced a burst of ROSs, whereas the upregulated expression of peroxidase, flavin-containing amine oxidoreductase and thiokinase was found in this study, suggesting the involvement of DAPs in free radical scavenging and antioxidant repair (**Figure 8**). It has been reported that peroxidases maintain cellular redox homeostasis, flavin-containing amine oxidoreductases regulate biogenic amine levels and thiolases are involved in cysteine metabolism or sulphur transport. This indicates their importance in antioxidant defence against freezing stress (Freitas et al., 2024; Akram et al., 2025; Göbbels et al., 2021). Transketolases are typically associated with photosynthesis and the pentose phosphate pathway, providing energy and metabolic support for plant adaptation (Sharkey, 2021). Therefore, we propose that DAPs in alfalfa are highly coordinated in response to freezing stress, involving the activation of the protein quality control system, enhancement of the antioxidant defense system and necessary metabolic adjustments.

### 4.6 Response of mRNA-protein pairs in alfalfa to freezing stress

Sugar metabolism provides the energy and material basis for plant stress tolerance and regulates the expression of stress tolerance genes via signaling networks (Kaur et al., 2021). Our analysis revealed that mRNA-protein pairs were highly enriched in the “Fructose and mannose metabolism” and “Galactose metabolism” pathways (**Figure 9**, **Supplementary Table S8**). This suggests that alfalfa shifts carbon sources from routine growth to the synthesis of key metabolites required for the stress response. Sorbitol dehydrogenase is an important osmoregulator that regulates sorbitol metabolism (Hu et al., 2023). Increasing sorbitol content can enrich carbohydrate metabolism and improve plant adaptation to adversity (Pleyerová et al., 2022). In this study, we found the upregulated expression of an mRNA-protein linkage pair encoding sorbitol dehydrogenase, which is included in the relevant pathway. This suggests that alfalfa accumulates sorbitol in order to regulate its response to freezing. In addition, we identified upregulated inducer response protein 1, which has been reported to be commonly involved in pathogen defense signaling (Liao et al., 2009). This implies that freezing stress triggers a defense mechanism similar to that of biotic stress. β-Galactosidase affects cell wall function by the regulation of degradation and modification of galactosylated polysaccharides in the plant (Paniagua et al., 2016). In this study, we identified a downregulated β-galactosidase 1, which was shown to be downregulated to maintain the integrity of the cell wall and protect against mechanical damage caused by excessive relaxation at low temperatures (**Figure 9**; **Supplementary Table S7**; **Supplementary Table S8**). We also found that related DEGs and DAPs were enriched in “Carbon metabolism”, “Glycolysis/Gluconeogenesis”, “Amino sugar and nucleotide sugar metabolism” pathways (**Figure 9**, **Supplementary Table S8**). This indirectly suggests that alfalfa synchronizes the maintenance of energy homeostasis with the strengthening of cellular structure through the regulation of carbon resources to enhance its viability in the freezing environment. Additionally, it has been demonstrated that ABA is a contributing factor to variations in fruit sugar content in apple (*Malus × domestica*). Furthermore, *MdWRKY9* synergistically regulates the expression of the sugar transporter protein *MdSWEET9b* alongside the abscisic acid signaling transporters *MdbZIP23* and *MdbZIP46*, thereby promoting fruit sugar accumulation (Zhang et al., 2023). Moreover, the expression of the β-galactosidase gene *FaβGal4* in strawberry (*Fragaria* x *ananassa*) can be activated by ABA (Paniagua et al., 2016). We have identified DEGs involved in the ABA pathway during freezing stress in alfalfa and concluded that these DEGs may enhance alfalfa’s freezing tolerance by modulating sugar metabolism responses.

The phenylpropane biosynthetic pathway begins with phenylalanine, which is catalyzed by key enzymes to produce a series of intermediates that ultimately form lignans, flavonoids, and anthocyanins (Liu et al., 2020). It has been observed that the phenylpropane pathway may contribute to enhanced pre-storage cold domestication and increased freezing tolerance in cucumber (*Cucumis sativus* L.) (Wang et al., 2021b). Peroxidase, a versatile enzyme commonly found in plants, can mitigate the oxidative damage caused by cold conditions by catalyzing the oxidation of H_2_O_2_ with substrates such as phenols and amines (El Zein et al., 2023). In this study, we identified a specific peroxidase family protein by finding that the mRNA-protein junction pair in the “Phenylpropanoid biosynthesis” pathway was upregulated (**Figure 9**, **Supplementary Table S8**). We propose that alfalfa activates antioxidant defense mechanisms to scavenge excess reactive oxygen species through the upregulation of peroxidase, thereby maintaining cellular homeostasis and protecting against freezing stress. Despite our integrated analysis, only a limited number of genes showed significant differential expression at both the mRNA and protein levels. This may be attributed to post-translational modifications and protein stability affecting protein levels. Future studies will involve processing and observing these genes at multiple time points.

This study reveals that alfalfa’s frost resistance involves multi-level regulation, inlcuding the transcriptional and protein levels. It is promising to develop superior cultivars with broad-spectrum, stable frost resistance through multi-parent hybridization or genetic engineering techniques. However, this study specifically analyzed only two contrasting cultivars subjected to short-term indoor freezing stress, in and the generalizability of the results requires further validation across a broader range of genetic materials and dynamic field environments. Future work will expand to multi-cultivar populations and incorporate field phenotypes for association analysis, aiming to distinguish core freeze-tolerance mechanisms from cultivar-specific responses.

## 5 Conclusions

In this study, we analyzed the physiological changes in two alfalfa cultivars by integrating proteomics with transcriptomics, focusing on DEGs and DAPs. Physiological analyses revealed higer activities of antioxidant enzymes and osmoregulatory substances levels in “Dongnong NO.1” than in “Bara 218TR”. Conversely, the MDA content and REL in “Dongnong NO.1” were lower than in “Bara 218TR”. Transcriptome analysis revealed that DEGs in both alfalfa species were predominantly enriched in the AP2/ERF-ERF transcription factor family. Multiple hormone signaling pathways within the “plant hormone signaling” pathway were activated under freezing stress. WGCNA analysis revealed that the expression of candidate genes associated with physiological phenotypes was influenced by freezing stress. The positive module responded to freezing stress by regulating calcium signaling via the activation of protein synthesis. Conversely, the negative module responded to freezing stress by inhibiting cell wall stiffening and mitigating iron toxicity. Proteome-based analysis indicated that DAPs were involved in protein quality control, the antioxidant defense system, and metabolic reprogramming. Furthermore, joint transcriptome and proteome analysis demonstrated that mRNA-protein linkage pairs were significantly enriched in pathways related to sugar metabolism, biotic stress-like defense, cell wall modification and phenylpropanoid biosynthesis. This suggests that these pathways play a critical role in alfalfa’s response to freezing stress, offering new insights into the underlying antifreeze mechanisms of this species.

## Data Availability

The datasets presented in this study can be found in online repositories. The names of the repository/repositories and accession number(s) can be found in the article/**Supplementary Material**.

## Supplementary material

Supplementary Figure 1 Alfalfa freezing stress treatment process.

Supplementary Table 1 Primers lists.

Supplementary Table 2 Clean data of 24 samples from GC and Q30 revised.

Supplementary Table 3 Results of clean data mapping to alfalfa reference genome.

Supplementary Table 4 Results of KEGG enrichment for the comparison of DEGs within and between two alfalfa cultivars.

Supplementary Table 5 Characterization of plant hormone pathway genes.

Supplementary Table 6 Forward and reverse module details in Cytoscape.

Supplementary Table 7 Junction pairs of mRNAs with the same or opposite expression trends as proteins.

Supplementary Table 8 Results of KEGG enrichment for the comparison of DEGs and DAPs within and between two alfalfa cultivars.
