# Supplementary material for: Combined transcriptomic and proteomic analysis reveals the response mechanisms of alfalfa to freezing stress
Source: Front Plant Sci. 2026 Jan 22;16:1682825. doi: 10.3389/fpls.2025.1682825 (PMC12872475; doi:10.3389/fpls.2025.1682825)
Supplement: Supplementary Figure 1 — Alfalfa freezing stress treatment process. [file DataSheet1.zip › Supplementary Materials/Figure S1.pdf]

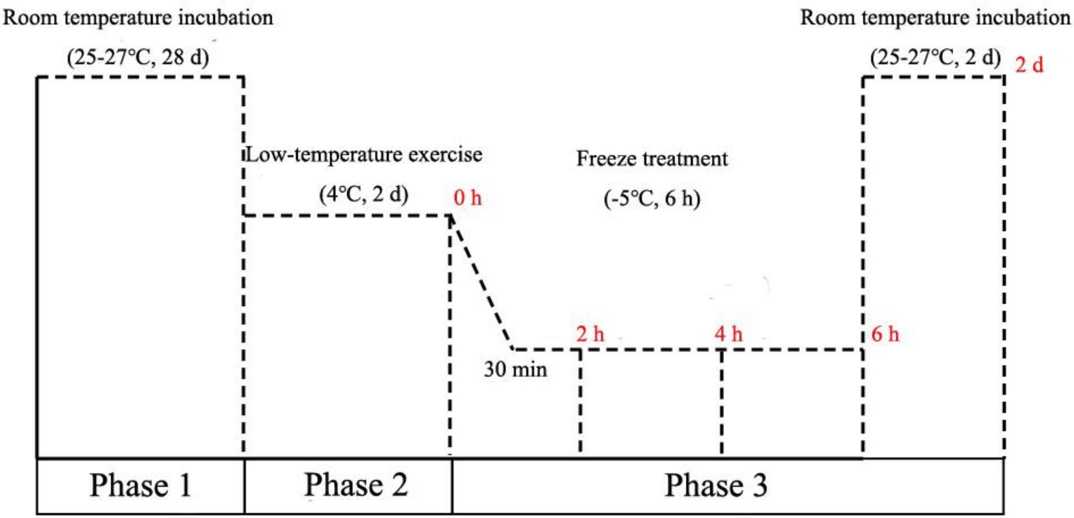

Supplementary Figure 1:Alfalfa freezing stress treatment process.
